# Me-LLaMA: Foundation Large Language Models for Medical Applications

**DOI:** 10.21203/rs.3.rs-4240043/v1

**Published:** 2024-05-22

**Authors:** Qianqian Xie, Qingyu Chen, Aokun Chen, Cheng Peng, Yan Hu, Fongci Lin, Xueqing Peng, Jimin Huang, Jeffrey Zhang, Vipina Keloth, Xinyu Zhou, Huan He, Lucila Ohno-Machado, Yonghui Wu, Hua Xu, Jiang Bian

**Affiliations:** 1 Section of Biomedical Informatics and Data Science, School of Medicine, Yale University, New Haven, CT, USA; 2 Department of Health Outcomes and Biomedical Informatics, College of Medicine, University of Florida, Gainesville, FL, USA; 3 School of Biomedical Informatics, University of Texas Health Science, Center at Houston, Houston, TX, USA

## Abstract

Recent advancements in large language models (LLMs) such as ChatGPT and LLaMA have hinted at their potential to revolutionize medical applications, yet their application in clinical settings often reveals limitations due to a lack of specialized training on medical-specific data. In response to this challenge, this study introduces Me-LLaMA, a novel medical LLM family that includes foundation models – Me-LLaMA 13/70B, along with their chat-enhanced versions – Me-LLaMA 13/70B-chat, developed through continual pre-training and instruction tuning of LLaMA2 using large medical datasets. Our methodology leverages a comprehensive domain-specific data suite, including a large-scale, continual pre-training dataset with 129B tokens, an instruction tuning dataset with 214k samples, and a new medical evaluation benchmark (MIBE) across six critical medical tasks with 12 datasets. Our extensive evaluation using the MIBE shows that Me-LLaMA models achieve overall better performance than existing open-source medical LLMs in zero-shot, few-shot and supervised learning abilities. With task-specific instruction tuning, Me-LLaMA models outperform ChatGPT on 7 out of 8 datasets and GPT-4 on 5 out of 8 datasets. In addition, we investigated the catastrophic forgetting problem, and our results show that Me-LLaMA models outperform other open-source medical LLMs in mitigating this issue. Me-LLaMA is one of the largest open-source medical foundation LLMs that use both biomedical and clinical data. It exhibits superior performance across both general and medical tasks compared to other open-source medical LLMs, rendering it an attractive choice for medical AI applications. We release our models, datasets, and evaluation scripts at: https://github.com/BIDS-Xu-Lab/Me-LLaMA.

## INTRODUCTION

In the quest to advance patient care and streamline clinical workflows, the emergence of large language models (LLMs) has marked a significant milestone.^1,2,3^ LLMs are language models^4^ with tens or hundreds of billions of parameters, trained on vast amounts of text data. They can generate human-level responses based on instructions, and perform complex tasks^1,5,6^ and have shown great potential in improving clinical documentation, diagnostic accuracy, and patient care management.^2,3,7,8^ However, a critical limitation of these LLMs, such as ChatGPT^9^ and GPT-4,^10^ is their closed-source nature.^11,12,13^ This limits extensive customization and accessibility, which are paramount in medical contexts requiring precise, context-specific applications.^14^

Addressing this challenge, recent research efforts have pivoted towards the development of open-source LLMs.^6^ These open-source alternatives offer a promising solution, facilitating unrestricted access and the flexibility of customizing these models to meet specific requirements of medical domain. LLaMA models^15,16^ is at the forefront of open-source LLMs in the general domain, exemplifying state-of-the-art (SOTA) capabilities. However, they still lack the specialized medical knowledge that are essential for accurate and reliable applications in medicine, since they are trained mainly on datasets from the general domain.^17,18^

To address these shortcomings, there has been a shift towards developing medical specific LLMs^11,12,13,19,20,21,22^ by enhancing open-source LLMs using biomedical data. However, existing studies such as PMC-LLaMA^11^ and Meditron^13^ only focus on the biomedical domain and evaluate only the question answering (QA) task. Very few of them used clinical data and evaluated clinical tasks where GatorTronGPT^21^ and Clinical-LLaMA^22^ are two exceptions. However, GatorTronGPT has limited in-context learning capabilities due to lacking instruction tuning, and limited model and data sizes, while Clinical-LLaMA’s limited clinical text pre-training and focus on classification tasks fail to fully exploit LLMs in varied clinical scenarios. Additionally, the development of these models faces the challenge of catastrophic forgetting, where integrating new medical data erodes prior knowledge, affecting performance across both general and specialized medical tasks.

To address the above challenges, we developed Me-LLaMA, a new family of medical LLMs, including foundation models (Me-LLaMA 13/70B), and their chat-enhanced versions (Me-LLaMA 13/70B-chat), developed through continual pre-training and instruction tuning of LLaMA2 models^16^ incorporating a rich diversity of biomedical and clinical data. We present a comprehensive data suite for medical LLMs research, including a large-scale, continual pre-training dataset with 129B tokens, an instruction tuning dataset with 214k samples, and a novel medical evaluation benchmark (MIBE) across six tasks with 12 datasets. Our extensive evaluation using the MIBE shows that Me-LLaMA models achieve overall better performance than existing open-source medical LLMs in zero-shot, few-shot and supervised learning abilities. With task-specific instruction tuning, Me-LLaMA models outperform ChatGPT on 7 out of 8 datasets and GPT-4 on 5 out of 8 datasets. This underscores the efficacy of our continual pre-training and instruction-tuning methodologies, and the robustness of our models in diverse medical application scenarios.

Our contributions can be summarized to the following:

- We propose a new medical foundation model family: Me-LLaMA 13B/70B encoding broad medical knowledge, and Me-LLaMA 13/70B-chat with superior instruction-following abilities. They are the first medical LLMs developed using both clinical and biomedical data. Our rigorous evaluations show that Me-LLaMA models outperform existing open-source medical LLMs in zero-shot, few-shot and supervised learning across different medical tasks.
- We propose a comprehensive data suite, including the largest continual pre-training data currently available, instruction tuning data, and the evaluation benchmark MIBE, spanning general, biomedical, and clinical domains. Our data suite is designed to address existing dataset gaps in medical LLM research, thereby accelerating advancements in medical LLMs by offering a robust data foundation for training and benchmarking.
- We investigate the catastrophic forgetting problem in existing medical LLMs, revealing a notable decline in both general and medical domain knowledge in these models. With optimization, Me-LLaMA models excel in mitigating the catastrophic forgetting problem, surpassing other medical LLMs in retaining prior knowledge across updates.

## RESULTS

### Overall Performance

Table 1 shows the zero-shot performance of Me-LLaMA chat models and baselines on various tasks in MIBE. Our baseline comparison includes LLMs that have undergone instruction fine-tuning to enhance their ability to follow instructions, such as the LLaMA2 chat models. Among models with 13B parameters, Me-LLaMA 13B-chat outperformed LLaMA2 13B-chat, PMC-LLaMA-chat, Medalpaca 13B in almost all 12 datasets, with the exception of a slight decrease in accuracy on the clinical question answering (QA) data EmrQA. Me-LLaMA outperformed AlpaCare-13B in 9 out of 12 datasets. Among models with 70B parameters, Me-LLaMA 70B-chat consistently outperformed Meditron 70B on all 12 datasets and achieved better performance than LLaMA2–70B-chat on 11 out of 12 datasets. Me-LLaMA 70B-chat outperformed the LLaMA2–70B-chat model by nearly 10% in accuracy and 8.0% in the Macro-F1 score on the PubMedQA dataset. It is worth noting that Me-LLaMA13B-chat showed better performance than LLaMA2–70B-chat—a model with a significantly larger parameter size—on 6 out of 12 datasets (including PubMedQA, MedQA, MedMCQA, 2013 DDI, HoC, MIMIC-CXR) and was competitive with the LLaMA2–70B-chat in 3 out of 6 remaining datasets (including EmrQA, MTsample, MedNLI).

**Figure 1.**
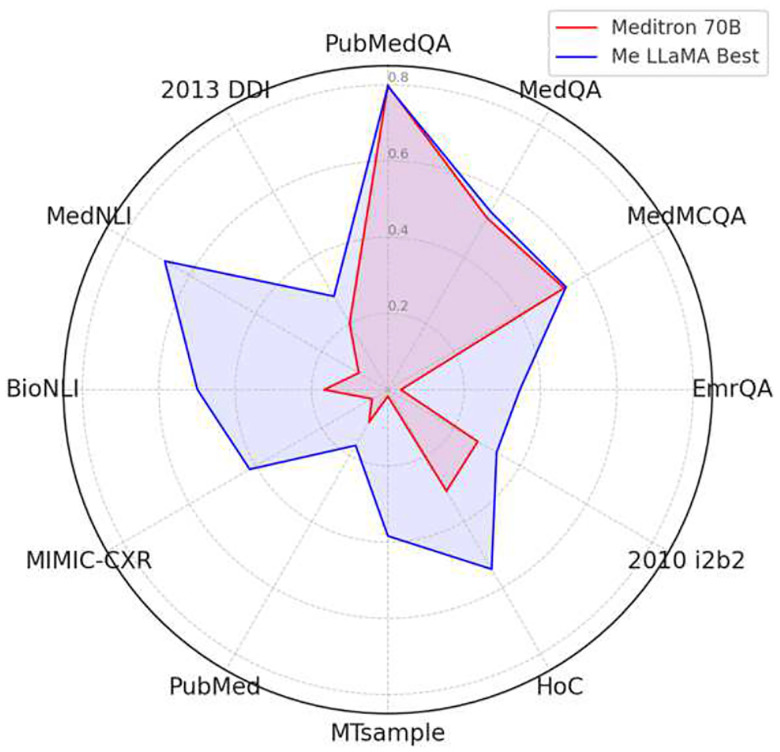
The comparison of the peak performance of Me-LLaMA models and Meditron 70B in the few-shot setting.

In Figure 1, we further compare the few-shot performance between Me-LLaMA models with Meditron 70B, the current SOTA medical LLM. We report the Rouge-L score for PubMed, the accuracy scores for three QA datasets, and the F1 scores for the remaining datasets. Given Meditron’s limited ability to follow instructions, our performance comparison utilizes a few-shot approach, employing a 1-shot method for summarization datasets due to their extensive input lengths and a 5-shot method for the rest. The 5-shot results of Meditron 70B on PubMedQA, MedQA and MedMCQA are referred from the first version of its paper.^13^ We can see that Me-LLaMA models achieved better performance in 11 out of 12 datasets (except for PubMedQA).

Table 2 compares the performance of our Me-LLaMA 13/70B foundation models against other open foundation LLMs in the supervised setting. We can observe that the Me-LLaMA 13B model surpassed the similar-sized medical foundation model PMC-LLaMA 13B on 11 out of 12 datasets and outperformed the general foundation model LLaMA2 13B on 10 out of 12 datasets, with the exception of DDI and HoC. Moreover, it is noticed that the Me-LLaMA 13B model was competitive with LLaMA2 70B and Meditron 70B, which have significantly larger parameter sizes, on 8 out of 12 datasets (PubMedQA, EmrQA, 2010 i2b2, MTsample, PubMed, MIMIC-CXR, BioNLI, and MedNLI). As for 70B models, Me-LLaMA 70B achieved the best performance on 9 out of 12 datasets (except for MedMCQA, 2010 i2b2 and PubMed), when benchmarked against LLaMA2 70B and Meditron 70B.

**Figure 2.**
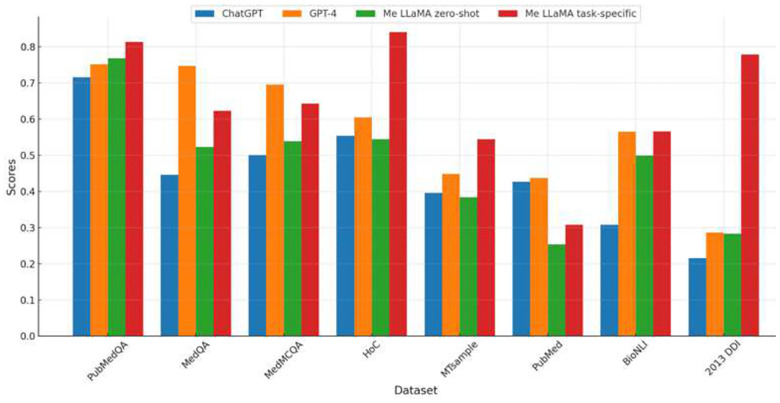
The comparison of performance of Me-LLaMA models in both zero-shot and task-specific instruction fine-tuning settings, against the zero-shot performance of ChatGPT and GPT-4.

Figure 2 further compares the performance of Me-LLaMA models in the zero-shot and task-specific instruction fine-tuning setting, against ChatGPT and GPT-4. Due to privacy concerns, which preclude the transmission of clinical datasets with patient information to ChatGPT and GPT-4, we conducted our comparison across eight datasets that are not subject to these limitations, including PubMedQA, MedQA, MedMCQA, HoC, MTsample, PubMed, BioNLI and 2013 DDI. The results of ChatGPT and GPT-4 on three QA datasets are referenced from the OpenAI’s paper.^3^ We compared the Rouge-1^27^ score for the summarization dataset PubMed, the accuracy score for three QA datasets, and the Macro-F1 score for the remaining datasets. With task-specific instruction tuning, Me-LLaMA models surpassed ChatGPT on 7 out of 8 datasets (except PubMed) and excelled GPT-4 on 5 out of 8 datasets, including PubMedQA, HoC, MTsample, BioNLI and 2013 DDI. In the zero-shot setting, Me-LLaMA models outperformed ChatGPT on 5 datasets (PubMedQA, MedQA, MedMCQA, BioNLI and 2013 DDI); but it fell short on 7 datasets, when compared with GPT-4. It’s crucial to highlight that Me-LLaMA’s model size is significantly smaller—13/70B parameters versus at least 175B for ChatGPT and GPT-4. Despite this size discrepancy, Me-LLaMA models have showcased an impressive performance and a strong ability for supervised learning and in-context learning across a broad spectrum of medical tasks, underscoring its efficiency and potential in the field.

### Impact of Continual Pretraining and Instruction Tuning

Table 3 compares the zero-shot performances of various LLMs to illustrate the impact of continual pre-training and instruction tuning. Specifically, this comparison focuses on the difference between Me-LLaMA 13/70B with their backbone models LLaMA2 13/70B in the zero-shot setting, highlighting the benefits of continual pre-training. Additionally, it examines Me-LLaMA 13/70B against their chat-optimized versions Me-LLaMA-13/70B-chat that received instruction tuning, showcasing the advantages of instruction tuning in zero-shot contexts.

Overall, Table 3 clearly demonstrates that both continual pre-training and instruction tuning significantly enhance the zero-shot capabilities of models. For example, the Me-LLaMA 13B model showed an improvement in performance ranging from 0.5% to 13.1% across various datasets in comparison to the LLaMA2 13B model, highlighting the benefits of continual pre-training. Compared with continual pre-training, instruction tuning was found to provide greater increases in zero-shot performance. For instance, the Me-LLaMA-70B-chat model displayed enhancements in performance from 3.7% to 41.9% relative to the Me-LLaMA 70B foundation model, which had not undergone instruction tuning. This enhancement suggests the critical role of instruction finetuning for boosting the model’s ability to leverage context in learning tasks, even without supervised fine-tuning and prior examples.

### Investigation of the Catastrophic Forgetting Problem

We conduct a comparison of existing medical LLMs to evaluate their vulnerability to catastrophic forgetting,^23,24,25^ where these LLM models forget old knowledge as they learn from new data. This issue is particularly critical for medical LLMs that need to maintain an accurate and consistent knowledge from both general and medical domains. Table 4 shows the performance comparison of various medical LLMs after continual pre-training against their backbone models on the general domain data MMLU^28^ and the medical data MedQA. Our Me-LLaMA models exhibited enhanced performance for both general and medical domains. In contrast, some models only showed improvements in medical data, with others experiencing performance declines in both domains after continual pre-training using medical data. Specifically, Meditron 7/70B showed improvements in the MedQA dataset but faltered on the MMLU dataset; whereas PMC-LLaMA 7/13B experienced declines in performance on both datasets. These outcomes underscore the significance of maintaining a balanced blend of general and medical data during training to prevent knowledge loss.

## DISCUSSION

### Model Performance

We introduced a novel medical LLM family including, Me-LLaMA 13B and Me-LLaMA 70B, which encode comprehensive medical knowledge, along with their chat-optimized variants: Me-LLaMA-13/70B-chat, with strong in-context learning ability, for medical applications. These models were developed through the continual pre-training and instruction tuning of LLaMA2 models, using a wide range of biomedical, clinical, and general domain data. We presented a comprehensive data suite including a large-scale, continual pre-training dataset with 129B tokens, an instruction tuning dataset with 214k samples, and a novel medical evaluation benchmark (MIBE) across six tasks with 12 datasets that span clinical, biomedical, and general domains, to support the training and evaluation of future medical LLMs. Our evaluations using the MIBE reveal that our Me-LLaMA models outperform existing open-source medical LLMs in various learning scenarios, showing less susceptibility to catastrophic forgetting and achieving competitive results against major commercial models including ChatGPT and GPT-4. Our work paves the way for more accurate, reliable, and comprehensive medical LLMs, and underscores the potential of LLMs on medical applications. We acknowledge that this is just a first step in the journey of providing patients and healthcare providers with a reliable source of medical knowledge, and our benchmark platform does not cover a large spectrum of cases so we can determine the absolute value of our models in real-world situations. However, in terms of relative value, we show that Me-LLaMA models compare favorably to their open-source counterparts.

In the zero-shot setting, medical LLMs including our models displayed low performance on certain tasks, e.g., NER and RE, which are also noted by other studies.^29,30^ When compared with other NLP tasks with higher performance, we noticed that one of the main reasons for low performance is that LLMs’ responses often lacked the conciseness and precision expected, with instances of missing outputs noted. For instance, within the zero-shot outputs of Me-LLaMA-13B-chat, challenges emerged across multiple tasks: In multi-label classification, the model frequently produced verbose sentences rather than the expected concise labels, occasionally offering slight label variations (e.g., “Avoid immune destruction” vs. “avoiding immune destruction”); for the NLI task, the outputs included zero-width spaces, incorrect numerical responses, and various unrelated strings; the model’s most significant struggle was with NER, where it frequently generated irrelevant responses do not present in the input sentence, indicating low instruction following capabilities. Therefore, more investigation is needed to further improve medical LLMs’ performance across tasks in the zero-shot setting. For example, one study proposed task-specific prompt frameworks to improve GPT-4’s performance on clinical NER tasks.^31^ Moreover, additional effort is required for enhancing the automatic assessment of these medical LLMs’ zero-shot capabilities. For instance, as indicated in Table 3, the zero-shot accuracy scores for LLaMA2 13B and Me-LLaMA 13B on MedQA were reported as 0. However, our manual evaluation revealed that the automatic evaluation metrics failed to capture their true performance accurately. This discrepancy is due to the models’ inability to adhere strictly to instructions and their generation of unexpected outputs, posing significant challenges to automatic evaluation metrics.

In the supervised finetuning setting, the Me-LLaMA models have demonstrated superior or comparable performance across most tasks when compared with SOTA methods before the LLM era. Previous methods have achieved the accuracy scores of 0.744 in PubMedQA,^32^ 0.503 in MedQA,^32^ 0.430 in MedMCQA,^33^ the macro-F1 score of 0.795 in EmrQA,^34^ 0.853 in the 2013 DDI,^35^ 0.800 in the 2010 i2b2,^33^ 0.864 in HoC,^36^ 0.650 in MTsample,^37^ 0.620 in BioNLI,^38^ 0.866 in MedNLI,^36^ the Rouge-L score of 0.503^39^ for PubMed and 0.471 for MIMIC-CXR.^40^ However, a notable exception is observed in the summarization dataset of PubMed, where their performance is significantly lower than those methods based on pre-trained language models like BART.^41^ This shortfall is linked to the poor quality of gold standard summaries in the dataset,^42^ which not only degrades the quality of model-generated summaries but also biases evaluation metrics. Furthermore, studies show that common automatic evaluation metrics, such as Rouge, fail to accurately reflect the quality of LLM summaries, which may receive lower automated scores despite high ratings from human evaluators.^42^

### Model Development

During our model development, we noticed the importance of diversity of the data sources during the pre-training and instruction-tuning phases. High-quality data, meticulously curated from a wide range of comprehensive sources, forms the cornerstone of our models’ performance, ensuring that the models can grasp broad medical and biomedical concepts with exceptional precision. Nevertheless, it is important to combine both medical and general domains’ data to ensure the robustness of the developed models. Our investigations revealed that the PMC-LLaMA 13B model, which employed a data mix ratio of 19:1 between medical and general domain data, exhibited diminished performance across both general and biomedical tasks. On the other hand, the Meditron models, 7B, and 70B, with a 99:1 mix ratio, demonstrated improvements in biomedical tasks, yet they still saw declines in the performance of general tasks. In contrast, our models, which adopt a 4:1 ratio, have shown enhancements in their performance for both general and medical tasks. This suggests that the integration of general domain data plays a vital role in mitigating the knowledge-forgetting issue during pre-training.^11,24,25^ However, determining the optimal balance between general domain data and specialized medical data is nontrivial, requiring careful empirical analysis. Future studies should examine methods to better determine the optimal ratio.

Our model development also underscores the balance between cost and effectiveness in pre-training versus instruction tuning of LLMs. Pre-training, exemplified by the LLaMA2 70B model, is notably resource-heavy, requiring about 700 hours on 160 A100 GPUs per epoch. Conversely, instruction tuning is far less resource-demanding, needing roughly 70 hours on 8 A100 GPUs per epoch, making it much more affordable than pre-training. Despite this, instruction tuning alone enhanced the performance of the Me-LLaMA-13B-chat model, achieving improvements ranging from 12% to 45% across 11 out of 12 datasets when compared to its backbone model – Me-LLaMA 13B, in the zero-shot setting. This efficiency advocates for prioritizing instruction tuning in scenarios with limited resources, highlighting its potential for cost-effective model enhancement.

### Use of Me-LLaMA Models

The Me-LLaMA models, available in both 13B and 70B sizes, as well as in base and chat-optimized versions, unlock a wide array of medical applications, guided by the crucial balance between model size and resource availability. The base models serve as robust foundations with extensive medical knowledge, adaptable through supervised fine-tuning for specialized tasks. Conversely, the chat versions excel in instruction-following ability and in-context learning, making them highly effective in zero-shot or few-shot learning scenarios. Larger models, like the 70B, provide deeper understanding and more complex reasoning abilities, ideal for comprehensive medical analyses. Yet, their deployment requires significant computing resources, posing challenges in resource-limited settings. On the other hand, the 13B models offer a practical compromise, balancing efficiency with effectiveness, thus ensuring broader accessibility for various applications. Our findings indicate that the Me-LLaMA 13B achieves performance on par with the 70B variant across most datasets, suggesting its viability for diverse medical tasks where computational or financial resources are a concern.

### Limitations

It is crucial to acknowledge the limitations of the current versions of Me-LLaMA models. Like all existing LLMs, they are susceptible to generating information with factual errors or biased information. To mitigate this, future studies could incorporate methodologies like reinforcement learning from human feedback (RLHF).^43^ This approach could align the models’ responses more closely with human values and ensure they are grounded in factual medical knowledge. Another limitation is the current token handling capacity, capped at 4096 tokens, which is a constraint inherited from the backbone LLaMA2 model. Addressing this limitation could involve extending the models’ capability to handle longer contexts. This could be achieved by integrating advanced attention techniques, such as sparse local attention,^44^ that are able to handle extensive contexts.

## METHODS

**Figure 3.**
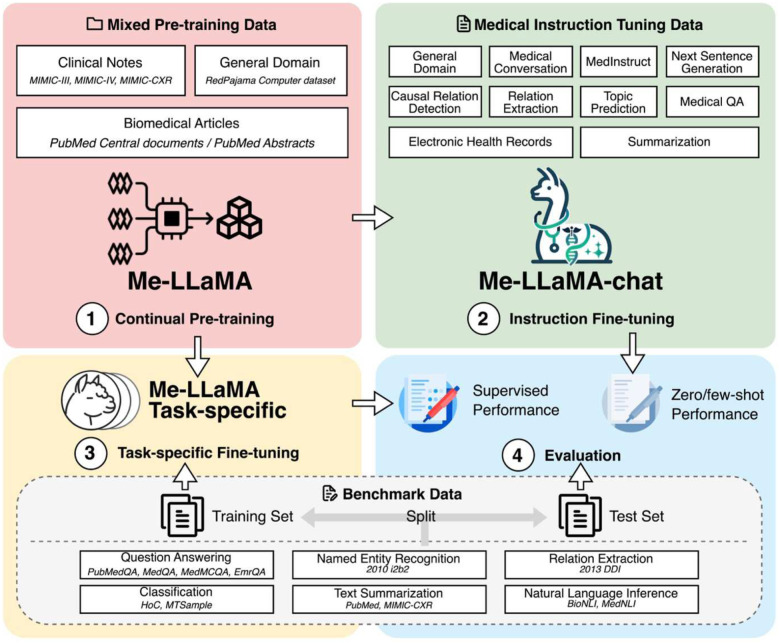
Overview of the study. We first developed the Me-LLaMA base models by continual pre-training LLaMA2 models with mixed pre-training data (stage 1). Me-LLaMA-chat models are further developed by instruction fine-tuning Me-LLaMA base models (stage 2). We further fine-tuned Me-LLaMA base models with the task-specific training sets of evaluation datasets (stage 3) to evaluate their performance in the supervised learning setting and evaluate the performance of Me-LLaMA-chat models in the zero/few-shot setting (stage 4).

We developed Me-LLaMA through the process of continual pre-training and instruction tuning of LLaMA2, which incorporates 129B tokens and 214K instruction tuning samples from general, biomedical, and clinical domains. Figure 3 shows an overview of our study.

### Continual Pre-Training Data

To effectively adapt backbone LLaMA2 models for the medical domain through continual pre-training, we develop a mixed continual pre-training dataset. This dataset with 129B tokens, comprised of biomedical literature, clinical notes, and general domain data, aiming to balance domain-specific knowledge with broader context understanding, thereby mitigating catastrophic forgetting.

Biomedical Papers: Our dataset integrates a vast collection of biomedical literature from PubMed Central and PubMed Abstracts, sourced from the Pile dataset.^45^

Clinical Notes: To incorporate real-world clinical scenarios and reasoning, we include de-identified free-text clinical notes from MIMIC-III,^46^ MIMIC-IV,^47^ and MIMIC-CXR.^48^

General Domain Data: To combat catastrophic forgetting, we incorporate a subset from the RedPajama^49^ dataset, a replication of LLaMA’s pre-training data. We use a 15:1:4 ratio for biomedical, clinical to general domain data. This ratio helps in maintaining a strong focus on the medical domain, while also incorporating a broad spectrum of general knowledge.

### Medical Instruction Tuning Data

To enhance our model’s ability to follow instructions and generalize across diverse medical tasks,^49,53^ we further develop a novel medical instruction tuning dataset. This dataset encompasses a wide array of data sources, including biomedical literature, clinical notes, clinical guidelines, wikidoc, knowledge graphs, and general domain data, as shown in Table 5. The diverse tasks included in this dataset aim to refine the model’s ability to process and respond to medical information accurately and contextually. Our medical instruction tuning dataset finally contains 214,595 high-quality samples after removing noise like null inputs and responses. Detailed prompts for each data and the data example are shown in Appendix 0.1, Table A.1.

### Training Details

We developed our Me-LLaMA models: Me-LLaMA 13B and Me-LLaMA 70B by continual pre-training and instruction tuning, leveraging the LLaMA2 13B and LLaMA2 70B models as our backbone.

### Continual Pretraining:

We continually pretrained the LLaMA2 13B and LLaMA2 70B models with the pre-training datasets we constructed. This phase aimed to adapt LLaMA2 models to better understand and generate text relevant to the medical context. The training involves sequences of medical texts, where the model learned to predict the next token *x*_*i+1*_ in a sequence {*x*_1_, *x*_2_, …, *x*_n_}, maximizing the likelihood $ℒ(\Theta )=\sum_{i=1}^{n-1}logP_{\Theta }\left(x_{i+1}|x_{1},x_{2},\cdots ,x_{i}\right)$, where Θ is the parameter set of LLaMA2 models. This training was executed on the University of Florida’s HiPerGator AI supercomputer with 160 A100 80GB GPUs. We employed the AdamW optimizer with hyperparameters set to *β*_1_ to 0.9 and *β*_2_ to 0.95, alongside a weight decay of 0.00001 and a learning rate of 8e-6. We used a cosine learning rate scheduler with a 0.05 warmup ratio for gradual adaptation to training complexity and bf16 precision for computational efficiency. Gradient accumulation was set to 16 steps, and training was limited to one epoch. We utilized DeepSpeed^59^ for model parallelism.

### Instruction fine-tuning:

Following the continual pretraining phase, we advanced the development of our Me-LLaMA models (13B and 70B) through instruction tuning, using the developed instruction tuning data. Executed using 8 A100 GPUs, the fine-tuning process was set to run for 3 epochs with a learning rate of 1e-5. We used a weight decay of 0.00001 and a warmup ratio of 0.01 for regularization and gradual learning rate increase. We utilized LoRA-based^60^ parameter-efficient fine-tuning.

### Task-specific fine-tuning:

To evaluate Me-LLaMA 13/70B foundation models’ performances in the supervised learning setting, we conducted the task-specific finetuning on Me-LLaMA models (Me-LLaMA task-specific) with training sets of all assessed datasets in Table 6. We employed the AdamW optimizer with hyperparameters set to *β*_1_ = 0.9 and *β*_2_ = 0.95. For datasets with fewer than 10,000 training samples, we fine-tuned the models for 5 epochs, while for larger datasets, the fine-tuning was conducted for 3 epochs. Additional parameters included a weight decay of 0.00001 and a warmup ratio of 0.01. A uniform learning rate of 1e-5 was used across all datasets.

### Medical Evaluation Benchmark

Existing studies^11,13,22^ in the medical domain have primarily focused on evaluating the QA task. In this study, we introduce a novel and extensive evaluation benchmark, encompassing six distinct tasks: QA, Named Entity Recognition (NER), Relation Extraction (RE), Classification (CF), Text Summarization (TS), and Natural Language Inference (NLI). These tasks collectively involve 12 datasets meticulously sourced from biomedical, and clinical domains, offering a broad spectrum for evaluation as shown in Table 6. We also include a general domain QA data MMLU, for evaluating models’ knowledge forgetting issue on the general domain knowledge.

### Evaluation Settings

We evaluated our medical LLMs in two evaluation settings including in-context learning (zero-shot and few-shot learning) and supervised learning to evaluate their performance and generalization ability across various tasks compared to baseline models.

### In-context Learning

We first assessed our Me-LLaMA 13/70B-chat models’ in-context learning capabilities, which are key for intuitive task understanding and response without specific prior training. We compared our models and baseline models’ zero-shot and few-shot (5-shot) learning performance, using standardized prompts for each dataset, as detailed in Table A.2 shown in Appendix 0.2. We compared Me-LLaMA 13/70B-chat models with the following baseline models: ChatGPT/GPT-4 9,10: Representing SOTA commercialized LLMs. We used the version of “gpt-3.5-turbo-0301” for ChatGPT, and the version of “gpt-4–0314” for GPT-4. LLaMA2–7B/13B/70B-chat^16^ models were adaptations of the LLaMA2 series, optimized for dialogue and conversational scenarios. Medalpaca-7B/13B^12^ models were based on LLaMA-7B/13B, and these LLMs were specifically fine-tuned for tasks in the medical domain. The PMC-LLaMA-13B-chat^11^ model is an instruction-tuned LLM based on PMC-LLaMA-13B, demonstrating enhanced abilities in processing, and responding to medical instructions and queries. The AlpaCare-13B^54^ model is specifically tailored for clinical tasks based on LLaMA-2 13B by instruction tuning. Meditron 70B^13^ is continually pre-trained with a mix of clinical guidelines, medical papers, abstracts, and a small proportion of general data based on LLaMA2 70B.

### Supervised Learning

In the supervised learning setting, we evaluate Me-LLaMA 13/70B foundation models’ performances adapted to downstream tasks. We assess the performance of Me-LLaMA task-specific models on the test set of each dataset in Table 6. Our baseline models including LLaMA2 Models (7B/13B/70B)^16^: These are SOTA open-sourced LLMs. PMC-LLaMA 13B^11^ is a biomedical LLM continually pre-trained on a large corpus of 4.8M biomedical papers and medical books. Meditron7B/70B^13^ is continually pre-trained medical LLMs based on LLaMA2–7B/70B, with a mix of clinical guidelines, medical papers, abstracts, and a small proportion of general data.

## Figures and Tables

**Table 1.** The zero-shot performance of various large language models.

Task	Dataset	Metric	LLaMA2–13B-chat	PMC-LLaMA-chat	Medalpaca-13B	AlpaCare-13B	Me-LLaMA 13B-chat	LLaMA2–70B-chat	Meditron 70B	Me-LLaMA 70B-chat
Question answering	PubMedQA	Accuracy	0.546	0.504	0.238	0.538	**0.700**	0.668	0.718	**0.768**
		Macro-F1	0.457	0.305	0.192	0.373	**0.504**	0.477	0.516	**0.557**
	MedQA	Accuracy	0.097	0.207	0.143	0.304	**0.427**	0.376	0.428	**0.523**
		Macro-F1	0.148	0.158	0.102	0.281	**0.422**	0.367	0.419	**0.521**
	MedMCQA	Accuracy	0.321	0.212	0.205	0.385	**0.449**	0.339	0.368	**0.539**
		Macro-F1	0.243	0.216	0.164	0.358	**0.440**	0.273	0.382	**0.538**
	EmrQA	Accuracy	0.001	**0.053**	0.000	0.001	0.048	0.050	0.000	**0.119**
		F1	0.098	0.304	0.040	0.198	**0.307**	0.251	0.000	**0.346**
Named entity recognition	2010 i2b2	Macro-F1	0.143	0.091	0.000	**0.173**	0.166	0.321	0.121	**0.329**
Relation extraction	2013 DDI	Macro-F1	0.090	0.147	0.058	0.110	**0.214**	0.087	0.176	**0.283**
Classification	HoC	Macro-F1	0.228	0.184	0.246	0.267	**0.335**	0.309	0.258	**0.544**
	MTsample	Macro-F1	0.133	0.083	0.003	**0.273**	0.229	0.254	0.142	**0.384**
Summarization	PubMed	Rouge-L	0.161	0.028	0.014	**0.167**	0.116	**0.192**	0.169	0.169
		BERTS*	0.671	0.128	0.117	**0.671**	0.445	**0.684**	0.658	0.678
	MIMIC-CXR	Rouge-L	0.144	0.139	0.010	0.134	**0.400**	0.131	0.060	**0.418**
		BERTS*	0.704	0.694	0.502	0.702	**0.797**	0.696	0.582	**0.787**
Natural language inference	BioNLI	Macro-F1	0.173	0.159	0.164	0.170	**0.195**	0.297	0.194	**0.436**
	MedNLI	Macro-F1	0.412	0.175	0.175	0.275	**0.472**	0.515	0.218	**0.675**

* BERTS: BERTScore.^26^

**Table 2.** The supervised fine-tuning performance of various large language models.

Task	Dataset	Metric	LLaMA2 13B	PMC-LLaMA 3B	Me-LLaMA 13B	LLaMA2 70B	Meditron 70B	Me-LLaMA 70B
Question answering	PubMedQA	Acc	0.800	0.778	**0.802**	0.800	0.800*	**0.814**
		Macro-F1	0.560	0.544	**0.562**	0.560	-	**0.572**
	MedQA	Acc	0.467	0.456	**0.493**	0.598	0.607*	**0.623**
		Macro-F1	0.465	0.454	**0.487**	0.595	-	**0.621**
	MedMCQA	Acc	0.527	0.548	**0.557**	0.626	**0.651** *	0.643
		Macro-F1	0.524	0.545	**0.551**	0.625	-	**0.640**
	EmrQA	Acc	0.789	0.810	**0.857**	0.847	0.850	**0.854**
		F1	0.730	0.738	**0.751**	0.751	0.751	**0.751**
Named entity recognition	2010 i2b2	Macro-F1	0.904	0.901	**0.906**	**0.913**	0.908	0.910
Relation extraction	2013 DDI	Macro-F1	**0.622**	0.622	0.559	0.746	0.737	**0.779**
Classification	HoC	Macro-F1	**0.696**	0.422	0.684	0.818	0.702	**0.841**
	MTsample	Macro-F1	0.430	0.345	**0.451**	0.458	0.284	**0.544**
Summarization	PubMed	R-L	0.191	0.091	**0.197**	**0.211**	0.197	0.209
		BERTS	0.663	0.516	**0.679**	0.689	0.677	**0.700**
	MIMIC-CXR	R-L	0.437	0.139	**0.453**	0.440	0.458	**0.476**
		BERTS	0.816	0.694	**0.821**	0.813	0.824	**0.828**
Natural language inference	BioNLI	Macro-F1	0.409	0.332	**0.447**	0.447	0.444	**0.566**
	MedNLI	Macro-F1	0.881	0.868	**0.903**	0.884	0.897	**0.916**

* The performance of Meditron 70B on the PubMedQA, MedQA, and MedMCQA datasets is cited from the first version of its paper, which reports solely the accuracy score.^13^ Despite their higher reported performances on these datasets achieved through chain-of-thought fine-tuning, we refer to their results obtained through the fine-tuning, without the use of chain-of-thought, to ensure a fair comparison.

**Table 3.** The comparison of zero-shot performances among Me-LLaMA models and LLaMA2 models.

Dataset	Metric	LLaMA 2 13B	Me-LLaMA 13B	Me-LLaMA-13B-chat	LLaMA 2 70B	Me-LLaMA 70B	Me-LLaMA-70B-chat
PubMedQA	Acc	0.216	0.266	**0.700**	0.132	0.682	**0.768**
	Macro-F1	0.177	0.250	**0.504**	0.152	0.520	**0.557**
MedQA	Acc	0.000	0.000	**0.427**	0.005	0.281	**0.523**
	Macro-F1	0.000	0.000	**0.422**	0.009	0.350	**0.521**
MedMCQA	Acc	0.003	0.003	**0.449**	0.012	0.447	**0.539**
	Macro-F1	0.006	0.005	**0.440**	0.024	0.396	**0.538**
EmrQA	Acc	0.000	0.005	**0.048**	0.000	0.021	**0.119**
	F1	0.038	0.122	**0.307**	0.000	0.172	**0.346**
2010 i2b2	Macro-F1	0.008	0.030	**0.263**	0.181	0.224	**0.329**
2013 DDI	Macro-F1	0.035	0.036	**0.214**	0.034	0.118	**0.283**
HoC	Macro-F1	0.253	0.210	**0.335**	0.255	0.252	**0.544**
MTsample	Macro-F1	0.042	0.072	**0.229**	0.066	0.226	**0.384**
PubMed	R-L	**0.170**	0.168	0.116	0.167	0.119	**0.169**
	BERTS	**0.654**	0.654	0.445	0.654	0.654	**0.678**
MIMIC-CXR	R-L	0.051	0.172	**0.400**	0.059	0.137	**0.418**
	BERTS	0.566	0.697	**0.797**	0.577	0.649	**0.787**
BioNLI	Macro-F1	0.109	0.060	**0.195**	0.285	**0.499**	0.436
MedNLI	Macro-F1	0.172	0.206	**0.472**	0.265	0.256	**0.675**

**Table 4.** The performance of various large language models on MMLU and MedQA.

Dataset	MMLU			MedQA		
Model	Accuracy	F1	Macro-F1	Accuracy	F1	Macro-F1
LLaMA 7B^15^	0.501	0.501	0.500	0.298	0.294	0.295
PMC-LLaMA 7B^11^	0.474 (−2.7%)	0.475 (−2.6%)	0.474 (−2.6%)	0.277 (−2.1%)	0.265 (−2.9%)	0.265 (−3.0%)
LLaMA2 7B^16^	0.540	0.540	0.539	0.428	0.427	0.425
Meditron 7B^13^	0.528 (−1.2%)	0.529 (−1.1%)	0.528 (−1.1%)	0.479 (+5.1%)*	-	-
LLaMA2 13B^16^	0.570	0.571	0.570	0.467	0.469	0.465
PMC-LLaMA 13B^11^	0.540 (−3.0%)	0.541 (−3.0%)	0.540 (−3.0%)	0.456 (−1.1%)	0.456 (−1.3%)	0.454 (−1.1%)
Me-LLaMA 13B	0.580 (+1.0%)	0.581 (+1.0%)	0.581 (+1.1%)	0.493 (+2.6%)	0.491 (+2.2%)	0.487 (+2.2%)
LLaMA 70B^24^	0.692	0.693	0.692	0.598	0.597	0.595
Meditron 70B^17^	0.678 (−1.4%)	0.678 (−1.5%)	0.677 (− 1.5%)	0.607 (+0.9%)*	-	-
Me-LLaMA 70B	0.712 (+2.0%)	0.713 (+2.0%)	0.712 (+2.0%)	0.623 (+2.5%)	0.624 (+2.7%)	0.621 (+2.6%)

* The performance of Meditron 7/70B on MedQA is cited from their original publication, which reports solely the accuracy score.^13^

**Table 5.** The overall instruction tuning dataset.

Task	Type	Source	Size	Copy right
General	Conversation	Alpaca^50^	20,000	CC-BY-NC 4.0
		Dolly^51^		CC-BY-SA-3.0
		ShareGPT^52^		Apache-2.0
Biomedical	Conversation	HealthCareMagic^53^	20,000	Reserved by HealthCareMagic and Icliniq
		Icliniq^53^		
	Instructions	MedInstruct^54^	52,000	CC BY-NC 4.0
	Question Answering	Medical Flash Cards^12^	34,000	No commercialized use
		MEDIQA^55^	2,220	CC BY 4.0
		MedicationQA^56^	690	CC BY 4.0
		LiveQA^57^	634	CC BY 4.0
		WikiDocPatient^12^	5,490	CC BY-SA 4.0
		GuidelineQA	2,000	Common Crawl (other)
	Summarization	PubMed Central	10,000	CC BY
	Next Sentence Generation	PubMed Central	20,000	CC BY
	Key words prediction	PubMed Central	10,000	CC BY
	Causal Relation Detection	PubMed^58^	2,450	CC BY
	Relation Extraction	UMLS knowledge graph^11^	10,000	Openrail
Clinical	QA, summarization, classification, mortality prediction	MIMIC-III,^46^ MIMIC-IV^47^	30,000	PhysioNet credentialed health data use agreement 1.5.0

**Table 6.** Details of data splits and evaluation metrics of each dataset in the evaluation benchmark.

Data	Task	Train	Valid	Test	Evaluation
PubMedQA*^61^	QA	211,269	-	500	Accuracy, Macro-F1
MedQA^62^	QA	10,178	1,272	1,273	Accuracy, Macro-F1
MedMCQA*^63^	QA	182,822	-	4,183	Accuracy, Macro-F1
EmrQA^64^	QA	122,326	30,581	26,804	Exact match, F1
MMLU^28^	QA	20,000	1,531	14,042	Accuracy, Macro-F1
2010 i2b2^65^	NER	6,0875	7,400	7,451	Entity-level Macro-F1
2013 DDI^66^	RE	18,779	7,244	5,761	Macro-F1
HoC^67^	CF	1,108	157	315	Label-wise Macro-F1
MTSample^37^	CF	4,999	500	999	Accuracy, Macro-F1
PubMed^68^	TS	117,108	6,658	6,631	Rouge, BERTScore
MIMIC-CXR^48^	TS	122,014	957	1,606	Rouge, BERTScore
BioNLI^38^	NLI	5,544	5,000	6,308	Accuracy, Macro-F1
MedNLI^69^	NLI	11,232	1,422	1,395	Accuracy, Macro-F1

* Following previous studies, the original validation sets of PubMedQA and MedMCQA have been used as the test sets, and therefore randomly sampled 10% of the training set as the validation set during our training process.

## Data Availability

All datasets employed in the continual pre-training process and evaluation are accessible from their original published venues. The PubMed Central and PubMed Abstracts subset from The Pile are available at https://huggingface.co/datasets/EleutherAI/pile. MIMIC-IV and MIMIC-CXR datasets can be accessed under the PhysioNet Credentialed Health Data Use Agreement 1.5.0 at https://physionet.org/content/mimic-iv-note/2.2/ and https://physionet.org/content/mimic-cxr/2.0.0/ respectively. The RedPajama data is open-released at https://huggingface.co/datasets/togethercomputer/RedPajama-Data-1. Alpaca data is openly released at: https://github.com/tatsu-lab/stanford_alpaca. Dolly data is openly released at: https://huggingface.co/datasets/databricks/databricks-dolly-15k. Share GPT data can be accessed at: https://huggingface.co/datasets/anon8231489123/ShareGPT_Vicuna_unfiltered. The clinical instruction tuning data based on MIMIC-IV and MIMIC-CXR can be accessed under the PhysioNet Credentialed Health Data Use Agreement 1.5.0 through: https://huggingface.co/clinicalnlplab. The Medical Flash Cards and wikidoc QA datasets can be accessed at https://huggingface.co/medalpaca. Other remaining instruction tuning data can be openly accessed at: https://huggingface.co/clinicalnlplab. Me-LLaMA 13B and Me-LLaMA 70B models can be accessed at https://huggingface.co/clinicalnlplab, subject to the completion of a credentialed health data use agreement.

## APPENDIX

### 1 Medical Instruction tuning Data

Table A1 shows the prompt used for each instruction tuning dataset.

### 2 Medical Evaluation Benchmark

Table A2 shows the prompt for each dataset used in the evaluation benchmark.

**Table A1.** The prompt for each dataset.

Data	Prompt
General domain data	“Below is an input that describes a task, maybe paired with a context that provides further information. Write a response that appropriately completes the request. INPUT:{Text} CONTEXT:{Text} OUTPUT:”
Medical conversation data	“Given a medical query, provide a concise and clear answer based on the patient’s description. INPUT: {text} OUTPUT:”
MedInstruct	“Below is an input that describes a medical task, maybe paired with a context that provides further input information. Write a response that appropriately completes the request. INPUT: {text} CONTEXT: {text} OUTPUT:”
Medical flash cards	“If you are a medical professional, answer this question truthfully. INPUT: {text} OUTPUT:”
MEDIQA	“Answer the input medical question based on the given context. INPUT: {text} CONTEXT: {text} OUTPUT:”
MedicationQA	“Answer this medical question truthfully. INPUT: {text} OUTPUT:”
LiveQA	“Given a medical query, provide a concise and clear answer based on the given details. INPUT: {text} OUTPUT:”
Patient Information QA	“Answer this medical question truthfully. INPUT: {text} OUTPUT:”
GuidelineQA	“If you are a medical professional, answer this question truthfully. INPUT: {text} OUTPUT:”
Summarization	“Given an abstract of a biomedical paper, generate the title. INPUT: {text} OUTPUT:”
Pubmed sentence generation	“The task is to generate the subsequent sentence in a piece of biomedical literature based on the existing content. INPUT: {text} OUTPUT:”
Guideline sentence generation	“Write the next part for a clinical guideline. You’re given a piece of the guideline, and your task is to continue it. The new part should match the style and detail of the original and be medically correct. INPUT: {text} OUTPUT:”
Summarization	“Given an abstract of a biomedical paper, generate the title. INPUT: {text} OUTPUT:”
Topic prediction	“The task is to assign MeSH (Medical Subject Headings) terms to a given piece of biomedical literature. The input is the title and abstract of a biomedical literature. The output is a list of applicable MeSH terms. INPUT: {text} OUTPUT:”
Causal relation detection	“For the following statement, determine whether it offers: 1) Strong Advice: The statement gives a clear and assertive recommendation or guidance, or 2) Weak Advice: The statement provides a suggestion or mild recommendation but is not very assertive, or 3) No Advice: The statement doesn’t offer any recommendation or guidance. INPUT: {text} OUTPUT:”
Relation extraction	“Given a medical question, provide the answer to determine the relation between two medical terms in the question. INPUT: {text} OUTPUT:”
MIMIC radiology	“The task is to generate the radiology impression based on radiology findings from a patient’s clinical note. The input is the radiology findings from a patient’s clinical note. Generate an impression accordingly. INPUT: {text} OUTPUT:”
MIMIC disease multiple-choice	“The task is to determine whether a patient suffers from certain diseases, and you need to choose the right answer from the choices. The input is the clinical note of a patient. Please determine which of the following disease(s) occurred during the patient’s hospital stay, according to the clinical note in the input: A:{text} B:{text} C:{text} D:{text} Output format: The output should be A, B, C, or D only. INPUT: {text} OUTPUT:”
MIMIC disease QA	“The task is to determine whether a patient suffers from certain diseases. The input is the clinical note of a patient. Please respond with all of the disease(s) that occurred during the hospital stay, according to the clinical note in the input. INPUT: {text} OUTPUT:”
MIMIC disease binary classification	“The task is to determine whether a patient suffers from certain diseases. The input is the clinical note of a patient. Please determine whether all of the following disease(s) occurred during the patient’s hospital stay: {text}. Answer with True or False only. INPUT: {text} OUTPUT:”
MIMIC mortality	“The task is to determine whether the patient died while in the hospital. The input is the clinical note of a patient. Using the information in the input, determine whether the patient died while in the hospital. INPUT: {text} OUTPUT:”
MIMIC manual QA	“The task is answering a question based on a clinical note in the input and your knowledge. The input is the clinical note of a patient. Please answer the question: {text} INPUT: {text} OUTPUT:”

**Table A2.** The prompt for each dataset in our evaluation benchmark.

Data	Prompt
PubMedQA	“Your task is to answer biomedical questions using the given abstract. Only output yes, no, or maybe as answer. INPUT:{Text} CONTEXT:{Text} OUTPUT:”
MedQA	“You are a medical doctor taking the US Medical Licensing Examination. You need to demonstrate your understanding of basic and clinical science, medical knowledge, and mechanisms underlying health, disease, patient care, and modes of therapy. Show your ability to apply the knowledge essential for medical practice. For the following multiple-choice question, select one correct answer from A to E. Base your answer on the current and standard practices referenced in medical guidelines. Question:{text} Options: {text} Answer:”
MedMCQA	“You are a medical doctor answering realworld medical entrance exam questions. Based on your understanding of basic and clinical science, medical knowledge, and mechanisms underlying health, disease, patient care, and modes of therapy, answer the following multiple-choice question. Select one correct answer from A to D. Base your answer on the current and standard practices referenced in medical guidelines. Question:{text} Options: {text} Answer:”
EmrQA	“Given a medical context and an open-ended question related to it, extract the relevant text segment from the context as an answer. Expected output: Only extract and return the text segment from the provided context that directly answers the question. Do not add any new words. Context:{text} Answer:”
2010i2b2	“In the sentence extracted from clinical narrative notes, identify all the clinically relevant entities, including problems, tests, and treatments. The required answer format is the same sentence with HTML <span> tags to mark up specific entities. Entity Markup Guide: Use <span class=““problem””> to denote a medical problem. Use <span class=““ treatment””> to denote a treatment. Use <span class=““test””> to denote a test Input Text: {text} Output Text:”
2013 DDI	“The task is to predict relationship between the given head entity labeled as @DRUG1$ and tail entity labeled as @DRUG2$ within a given sentence, this relation which must be in (‘mechanism’, ‘effect’, ‘advice’, ‘int’, ‘none’). mechanism: this type is used to annotate drug-drug interactions that are described by their pharmacokinetic mechanism. effect: this type is used to annotate drug-drug interactions describing an effect or a pharmacodynamic mechanism. advice: this type is used when a recommendation or advice regarding a drug interaction is given. int: this type is used when a drug-drug interactions appears in the text without providing any additional information. none: there has no drug-drug interactions. INPUT: {text}. OUTPUT:”
HoC	“The task is to decide the Hallmarks of Cancer (HOC) taxonomy of the article based on its abstract. The input is an abstract text. There are 10 topics you will need to decide whether the article is related to. Topics: sustaining proliferative signaling, evading growth suppressors, resisting cell death, enabling replicative immortality, inducing angiogenesis, activating invasion and metastasis, genomic instability and mutation, tumor promoting inflammation, cellular energetics, avoiding immune destruction. The output should be topics from the above 10 topics, that are related to the input article. Please note one article can be related to multiple topics. Output format: provide a semicolon-separated list of relevant topics. INPUT:{text} OUTPUT:”
MTSample	“TASK: The task is to determine the medical specialty or domain that a medical transcription belongs to. The input is a medical transcription. There are 40 medical specialties or domains, and you need to decide which one is the transcription related to. The medical specialties or domains are: ‘Surgery’, ‘Allergy / Immunology’, ‘Sleep Medicine’, ‘Pediatrics - Neonatal’, ‘SOAP / Chart / Progress Notes’, ‘Bariatrics’, ‘Pain Management’, ‘Lab Medicine - Pathology’, ‘Dermatology’, ‘Orthopedic’, ‘Dentistry’, ‘Psychiatry / Psychology’, ‘General Medicine’, ‘Office Notes’, ‘Letters’, ‘Neurosurgery’, ‘Radiology’, ‘Cosmetic / Plastic Surgery’, ‘Nephrology’, ‘Diets and Nutritions’, ‘Chiropractic’, ‘Gastroenterology’, ‘Cardiovascular / Pulmonary’, ‘Speech - Language’, ‘Hospice - Palliative Care’, ‘Autopsy’, ‘Endocrinology’, ‘Emergency Room Reports’, ‘Discharge Summary’, ‘ENT - Otolaryngology’, ‘Urology’, ‘Physical Medicine - Rehab’, ‘Neurology’, ‘Podiatry’, ‘Ophthalmology’, ‘Rheumatology’, ‘IME-QME-Work Comp etc.’, ‘Hematology - Oncology’, ‘Consult - History and Phy.’, ‘Obstetrics / Gynecology’. The output should be only one medical specialty or domain from the above 40 specialties or domains, that is most relevant to the medical transcription. Please note that each medical transcript can only be related to one medical specialty or domain. Output format: provide the name of the medical specialty or domain. INPUT:{text} OUTPUT:”
PubMedSum	“The task is to summarize an input biomedical literature in six sentences. The input is a biomedical literature. The output is the summary of an input biomedical literature in six sentences. INPUT:{text} OUTPUT:”
MIMIC-CXR	“Derive the impression from findings in the radiology report. INPUT: {text} OUTPUT:”
BioNLI	“TASK: Please classify the relationship between the given premise and hypothesis into one of the following labels: entailment, contradiction, or neutral. Return only the label. INPUT:{text} OUTPUT:”
MedNLI	“TASK: Please classify the relationship between the given premise and hypothesis into one of the following labels: entailment, contradiction, or neutral. Return only the label. INPUT:{text} OUTPUT:”
